# Acridone Derivative 8a Induces Oxidative Stress-Mediated Apoptosis in CCRF-CEM Leukemia Cells: Application of Metabolomics in Mechanistic Studies of Antitumor Agents

**DOI:** 10.1371/journal.pone.0063572

**Published:** 2013-05-07

**Authors:** Yini Wang, Dan Gao, Zhe Chen, Shangfu Li, Chunmei Gao, Deliang Cao, Feng Liu, Hongxia Liu, Yuyang Jiang

**Affiliations:** 1 Department of Chemistry, Tsinghua University, Beijing, China; 2 The Key Laboratory of Tumor Metabolomics at Shenzhen, Shenzhen, China; 3 The State Key Laboratory Breeding Base-Shenzhen Key Laboratory of Chemical Biology, Graduate School at Shenzhen, Tsinghua University, Shenzhen, China; 4 School of Medicine, Tsinghua University, Beijing, China; Imperial College London, United Kingdom

## Abstract

A new acridone derivative, 2-aminoacetamido-10-(3, 5-dimethoxy)-benzyl-9(10H)-acridone hydrochloride (named 8a) synthesized in our lab shows potent antitumor activity, but the mechanism of action remains unclear. Herein, we report the use of an UPLC/Q-TOF MS metabolomic approach to study the effects of three compounds with structures optimized step-by-step, 9(10H)-acridone (A), 10-(3,5-dimethoxy)benzyl-9(10H)-acridone (I), and 8a, on CCRF-CEM leukemia cells and to shed new light on the probable antitumor mechanism of 8a. Acquired data were processed by principal component analysis (PCA) and orthogonal partial least squares discriminant analysis (OPLS-DA) to identify potential biomarkers. Comparing 8a-treated CCRF-CEM leukemia cells with vehicle control (DMSO), 23 distinct metabolites involved in five metabolic pathways were identified. Metabolites from glutathione (GSH) and glycerophospholipid metabolism were investigated in detail, and results showed that GSH level and the reduced/oxidized glutathione (GSH/GSSG) ratio were significantly decreased in 8a-treated cells, while L-cysteinyl-glycine (L-Cys-Gly) and glutamate were greatly increased. In glycerophospholipid metabolism, cell membrane components phosphatidylcholines (PCs) were decreased in 8a-treated cells, while the oxidative products lysophosphatidylcholines (LPCs) were significantly increased. We further found that in 8a-treated cells, the reactive oxygen species (ROS) and lipid peroxidation product malondialdehyde (MDA) were notably increased, accompanied with decrease of mitochondrial transmembrane potential, release of cytochrome C and activation of caspase-3. Taken together our results suggest that the acridone derivative 8a induces oxidative stress-mediated apoptosis in CCRF-CEM leukemia cells. The UPLC/Q-TOF MS based metabolomic approach provides novel insights into the mechanistic studies of antitumor drugs from a point distinct from traditional biological investigations.

## Introduction

Acridone derivatives with a unique molecular structure of two benzene rings fused together possess a wide range of biological activities, such as antivirus [1], anti-allergy [2], anti-malaria [3] and antitumor [4], [5], [6]. This feature is attributed to the semiplanar heterocyclic structure which appreciably interacts with different biomolecular targets. Acridone derivatives are found in natural plants [7]. However, the antitumor activity of acridone derivatives has attracted an increasing interest, and a large number of acridone derivatives have been chemically synthesized and tested for antitumor activity. These novel acridone derivatives act as DNA intercalators [8], [9], topoisomerase inhibitors [10], [11] or selective telomeric G-quadruplex DNA ligands [12], [13]. Antitumor activities of acridone derivatives have been traditionally characterized by molecular biological technologies [14], [15], [16], including MTT reduction assay for cellular proliferation, flow cytometry for cellular apoptosis, RT-PCR for gene expression, and Western blot for protein expression. The understanding of intensive antitumor mechanisms is limited because of the limitations of these conventional molecular biology approaches, such as poor repeatability, less quantity of data, and time-consuming and laboursome.

Metabolomics, a top-down systemic biology approach, provides insights into the global metabolic status of the entire organism through non-targeted analysis of metabolites in biological samples [17]. Moreover, metabolite alterations are not only sensitive to external stimuli and environmental factors but also respond to potential changes suggested by genomics and proteomics [18], [19]. Metabolomics has now been developed as a powerful tool for the disease diagnosis and monitoring [20], drug effect and toxicity evaluation [21], [22], biomarker identification [23], and food safety investigation [24], [25]. Therefore, metabolic profiling of cells underlying treatment with drug candidates is greatly helpful for understanding their action mechanisms and aids in their further modifications. Currently, the metabolomic-based technology has been reported for the exploration of drug-cell interactions [26], [27] and action mechanisms of drugs [28]. However, a comprehensive metabolite profile of acridone derivatives against cell lines is still lacking.

The metabolomic approach emphasizes on unbiased identification and quantification of low molecular weight metabolites (i.e. <1.5 kDa) present in biological samples, such as urine, plasma and cell extracts. Different analytical techniques have been used for metabolomic study, including gas chromatography-mass spectrometry (GC-MS) [29], liquid chromatography-mass spectrometry (LC-MS) [30] and nuclear magnetic resonance (NMR) [31]. Chemometric and mathematical modeling methods, such as principal component analysis (PCA) and orthogonal partial least squares discriminant analysis (OPLS-DA), are used to interpret the acquired complex data. Compared with LC-MS, ultra-performance liquid chromatography coupled to quadrupole time-of-flight mass spectrometry (UPLC-MS) offers several advantages, such as better chromatographic peak resolution, shorter analysis time and higher sensitivity, and thus is more appropriate for the metabolomic analysis.

**Figure 1:**
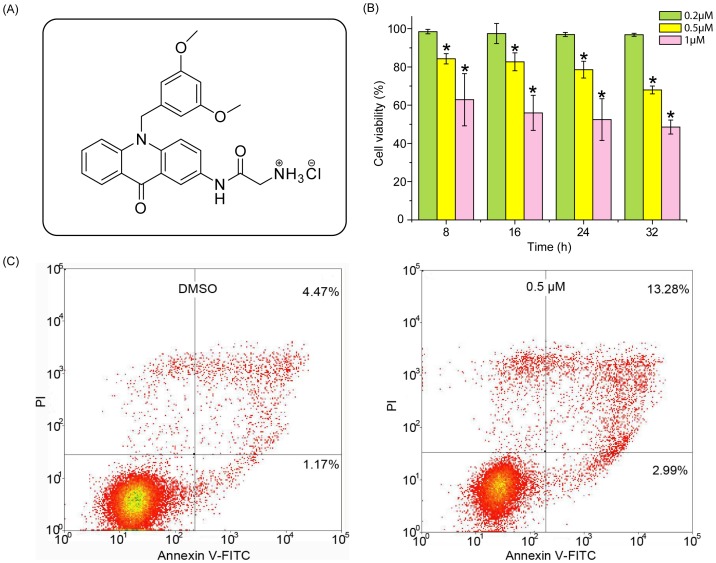
Chemical structure and antiproliferative activity against CCRF-CEM cells of acridone derivative 2-aminoacetamido-10-(3, 5-dimethoxy)-benzyl-9(10H)-acridone hydrochloride (8a). (A) Chemical structure of 8a. (B) Viability of CCRF-CEM cells after 24 h of exposure to 8a. The viability of the control cells, which were exposed to DMSO only, was set as 100%. n = 6, *p<0.05 compared with vehicle control. (C) Flow cytometric analysis of phosphatidylserine externalization (annexin V-binding) and cell membrane integrity (PI staining). CCRF-CEM cells were treated with 8a at 0.5 µM for 24 h.

In our previous studies, a series of acridone derivatives have been synthesized, and among them, the derivative 8a, 2-aminoacetamido-10-(3,5-dimethoxy)-benzyl-9(10H)-acridone hydrochloride (Figure 1A), has shown potent antitumor activity. However, the underlying mechanism of the anti-proliferative activity of 8a is unclear [32]. This current study presented a global analysis of metabolic changes in CCRF-CEM leukemia cells treated with 9(10H)-acridone (A), 10-(3,5-dimethoxy)benzyl-9(10H)-acridone (I), and 8a (the optimized structure based on the former two compounds). Distinct metabolites were identified and the involved biological events were verified by conventional molecular biology.

## Experiments

### Chemicals and Reagents

Compound 8a and I synthesized by our lab [33] and A (Sigma-Aldrich Co., USA) were dissolved in dimethyl sulfoxide (DMSO, Sigma-Aldrich Co., USA) with final concentration of 10 mM as stock solutions. Acetonitrile and methanol (HPLC grade) were purchased from Fisher (Fairlawn, USA). HPLC grade formic acid was purchased from Tedia (Tedia Co., USA). Distilled water was filtered through a Milli-Q system (Millipore, USA). RPMI 1640 was purchased from Invitrogen (Grand Island, USA). Fetal bovine serum (FBS) was purchased from Hyclone (Logan, USA). Streptomycin sulfate and penicillin were purchased from North China Pharmaceutical, China.

### Sample Preparation for Metabolomics

CCRF-CEM leukemia cells (Human T cell lymphoblast-like cell line) were obtained from the Chinese Academy of Sciences Cell Bank. The cells were cultured in RPMI 1640 medium supplemented with 10% FBS, 100 µg/mL penicillin, and 100 µg/mL streptomycin. Cells were maintained at 37°C in an atmosphere of 5% CO_2_ incubator with the medium replaced every 24 h. 1×10^7^ cells in a 10 cm dish were exposed to 0.5 µM of 8a, I or A or an equal amount of DMSO as a control. Eight replicates in separate dishes for each group were analyzed. The final concentration of DMSO was less than 0.1%. After 24 h incubation, cells were centrifuged at 300 *g* for 5 min at room temperature and harvested. The cell numbers were then counted with hemacytometer and used for normalization of the metabolite levels. The cell pellets were subsequently gentle resuspended in 1.0 mL ice cold isotonic saline (0.9% [w/v] NaCl) [34]. As reported in the literature, the cold isotonic saline did no damage cells and could effectively arrest cellular metabolism. The cells were then centrifuged at 1000 *g* for 1 min, and the isotonic saline solution was aspirated. The cell pellets were then immediately dissolved in 1.0 mL mixture of methanol/water in ratio of 4∶1 (v/v) at –20°C [35]. Then the cells were ultrasonicated in an ice bath ultra-sonicator for 10 min and subsequently centrifuged at 13000 *g* for 10 min at 4°C. The supernatant was collected and dried with a stream of nitrogen. The residues were resuspended in 1.0 mL acetonitrile/water (1∶1, v/v) mix, and were filtered through 0.22 µm mesh millipore filters (Florham Park, NJ) into glass auto-samplers. The samples were stored at –80°C prior to analysis. In parallel a quality control (QC) sample was prepared by mixing equal volumes of 30 µL into a glass auto-sampler from each of the 32 samples. The pooled QC samples were injected five times at the beginning and of the run in order to condition or equilibrate the system and then analyzed every three samples to further monitor the stability of the analysis.

### UPLC/Q-TOF MS Conditions

UPLC/Q-TOF MS analysis was performed using Acquity™ Ultra-Performance Liquid Chromatography system (Waters Corporation, MA, USA) coupled to Q-TOF premier Mass Spectrometer (Waters Corporation, MA, USA). The chromatographic separation was carried out on a Waters Acquity™ BEH C18 column (100 mm×2.1 mm, 1.7 µm). The column and samples were maintained at temperatures of 35°C and 4°C, respectively. The flow rate was at 0.5 mL/min. The mobile phase consisted of (A) 0.1% formic acid in water and (B) acetonitrile in positive mode, while (A1) 5 mM ammonium acetate in water and (B1) 5 mM ammonium acetate in acetonitrile (95∶5, v/v) in negative mode. Elution gradient was linearly increased from 5% to 40% B (B1) within 5 min, then to 100% B (B1) within 5 min and held for 1 min, followed by return to 5% B. Total running time was 14 min per separation. A 10 µL sample volume was introduced onto the column. The Waters Q-TOF premier equipped with an electrospray ion source in both positive and negative ion electrospray (ESI+ and ESI−) modes and V optics mode. The capillary voltage was 3.0 kV (ESI+) and 2.5 kV (ESI−), cone voltage was 35V (ESI+) and 30 V (ESI−). Cone gas flow was set at 50 L/hr with source temperature of 120°C. Desolvation gas flow was maintained at 550 L/hr with the desolvation gas temperature of 300°C. Data were collected in the centroid mode. The mass range was *m/z* 100–1000 with a scan time of 0.15 s and interscan time 0.02 s. For mass accuracy a LockSpray™ interface was used with Leucine-enkephalin (*m/z* 556.2771, ESI+; *m/z* 554.2615, ESI−) at a final concentration of 100 pg/uL in acetonitrile-water with 0.1% formic acid (50∶50 v/v) was used as the lock mass (*m/z* 556.2771) with a flow rate of 0.05 mL/min. Lock spray frequency was set at 10 s and scan to average for correction was 10 s with the reference cone voltage at 35 V. For MS/MS analysis, the collision energies were set in ramp mode ranged from 10 V to 30 V.

### Multivariate Data Analysis

Original data was processed using the MarkerLynx software (version 4.1, Waters Corporation, MA, USA) which used Apex-Track-peak detection package to integrate peaks in UPLC/MS data. Peaks in each chromatogram were identified by *m/z*, retention time (RT) as well as their associated height intensities. The three-dimensional data, peak number (RT_*m/z* pair), sample name, and normalized ion intensity were introduced to SIMCA-P 11.5 software package (Umetrics, Umea, Sweden) for multivariate data analysis. The variables were transformed by mean-centering and Pareto scaling to increase the low abundance ions without significant amplification of noise, and then analyzed by PCA and OPLS-DA. PCA is an unsupervised multivariate statistical approach. It is used for variable reduction and separation into classes. To maximize class discrimination and biomarkers, the data were further analyzed using the OPLS-DA method [36]. In this study, the control and 8a-treated groups were compared in the OPLS-DA method. S-plots were calculated to visualize the relationship between covariance and correlation within the OPLS-DA results. Variables that had significant contributions to discrimination between groups were considered as potential biomarkers and subjected to further identification of the molecular formula. Databases of HMDB (http://www.hmdb.ca/) and METLIN (http://metlin.scripps.edu/) were used to identify the metabolite markers with tandem mass spectrometry. Standards of metabolic interest were used to confirm their structures.

### Cell Viability and Flow Cytometric Assays

CCRF-CEM cells were suspended at a concentration of 2×10^5^ cells/mL and seeded in 96-well microtiter plates. 0.2, 0.5, and 1 µM of 8a were added to each well in sextuplet and then incubated for 8 h, 16 h, 24 h, and 32 h respectively. After treatment, the cells were incubated with 10 µL of MTT (3-(4,5-dimethyl-thiazol-2-yl)-2,5-diphenyl-tetrazolium bromide from Sigma) solution (5 mg/mL) for 4 h. The formazan precipitate was dissolved in 100 µL DMSO and the absorbance at 490 nm was measured by a Benchmark microplate reader (Molecular Devices Corporation, USA). The apoptotic cells were measured by the Annexin V-FITC/PI apoptosis detection kit (Beyotime Institute of Biotechnology, China) according to the protocol described using flow cytometry (Moflo XDP, Beckman Coulter, USA). Briefly, CCRF-CEM cells were exposed to 0.5 µM 8a for 24 h at 37°C. Then cells were collected by centrifugation and washed twice with ice-cold phosphate-buffered saline (PBS). Positive Annexin V staining indicated apoptosis, while positive PI indicated necrosis. For each group, a minimum of 10,000 cells were used.

### Detection of Reactive Oxygen Species (ROS) Level and Mitochondrial Transmembrane Potential (MMP)

The levels of intracellular ROS and MMP were determined according to their described protocol respectively. Cells were treated with 0.5, 1, 2 µM of 8a, harvested, and suspended in PBS. 1×10^6^ cells were separately incubated with 10 µM 2′, 7′-dichlorfluorescein-diacetate (DCFH-DA) and 2 µM Rh123 in dark for 30 min at 37°C for ROS and MMP detection. The fluorescence was detected with a fluorescence spectrophotometer (ROS, 485 nm Ex and 525 nm Em; MMP, 507 nm Ex and 529 nm Em).

### Determination of Lipid Peroxidation

Malondialdehyde (MDA), an indicator of lipid peroxidation was quantified by a Lipid Peroxidation MDA assay kit (Beyotime Institute of Biotechnology, China) according to the manufacturer’s protocol. In brief, cells were treated with 0.5, 1, 2 µM of 8a. They were centrifuged and sonicated in 100 µL of lysis buffer (10 mM Hepes pH 7.9, 10 mM KCl, 1 mM EDTA, 0.1% NP-40, 1 mM Na_3_VO_4_, 1 mM DTT) combined with proteinase inhibitor cocktail (Thermo Scientific, USA). After sonication, lysed cells were centrifuged at 10000 *g* for 10 min to remove debris. The supernatant was subjected to the measurement of malondialdehyde (MDA) levels and the protein contents. 100 µL of 0.09% Thiobarbituric acid (TBA) containing antioxidant was added to the supernatant to react with MDA. This mixture was incubated at 100°C for 15 min. Thiobarbituric acid reactive species (TBARS) produced were measured at 532 nm using Varioskan Flash Multimode Reader (Thermo Scientific, USA) and the absorbance was compared with that of standard curve using MDA. Protein concentrations were determined by BCA kit (Bio Tec Inc, USA) using a multi-function detector (Beckman, German). MDA levels were then normalized to milligram protein. The extraction and quantification of proteins were determined used the same procedure in the following assays unless otherwise indicated.

### Mitochondrial and Cytosolic Fractionation

Prior to determine the release of cytochrome C from mitochondria to cytosol, the isolation of mitochondria and cytosol was performed using the Cell Mitochondria Isolation Kit (Beyotime Institute of Biotechnology, China). Briefly, 5×10^7^ cells were treated with 0.5 µM of 8a and harvested. They were then incubated in 100 µL ice-cold mitochondrial lysis buffer on ice for 10 min. Cell suspension was then taken into a glass homogenizer and homogenized for 50 strokes using a tight pestle on ice. The homogenate was then centrifuged at 600 *g* for 10 min at 4°C to remove nuclei and unbroken cells. Then the supernatant was collected and centrifuged again at 12000 *g* for 15 min at 4°C to obtain the mitochondria (deposition) and cytosol (supernatant) fractions. Samples of mitochondria and cytosol were dissolved in lysis buffer and proteins were subjected to Western blotting, respectively.

### Western Blotting Analysis

Cells were treated with 0.5, 1 µM of 8a and harvested. Proteins were extracted from the cells, separated in 15% SDS-polyacrylamide gel, and then transferred to PVDF membranes. The membranes were blocked in TBST buffer (0.125 M NaCl,25 mM Tris pH 8.0) containing 5% defatted milk for 1 h and then incubated with specific first antibody overnight at 4°C. Protein bands were detected using the SuperSignal West Pico Chemiluminescent Substrate (Thermo Scientific, USA) after hybridization with horseradish peroxidase-conjugated second antibody. Procaspase-3 and cleaved caspase-3 antibodies (Cell Signaling Technologies, 9662) were the first antibodies for caspase detection, while anticytochrome C antibody (Abcam, ab76237) was for cytochrome C assay.

### Statistical Analysis

For metabolomic experiments, eight independent biological samples from separated dishes for each group were performed. While the biological experiments for the biological events verification were repeated at least three times. Data are expressed as means ± standard deviation (SD). Student’s t-test was used to determine the significances of differences in multiple comparisons. Values of *p*<0.05 were considered statistically significant.

## Results and Discussion

### Anti-proliferative Activity of Acridone Derivatives

Parental compound A had no cytotoxicity (IC_50_>100 µM), but the derivative compound I with dimethoxy groups on the benzene ring of A showed appreciable cell growth inhibition. Interestingly, the new derivative compound 8a with terminal amino substituents at C2 position on the acridone ring of I exhibited the most potent anti-proliferative activity with an IC_50_ at 0.39 µM (Table S1). Structure–activity analysis indicated that both the acridone group and substituted groups on the parental compound affected the cytotoxicity of acridone derivatives.

The non-targeted metabolomic approach as an emerging tool in cancer research was conducted in an unbiased manner. Prior to metabolomic analysis, the effects of compound concentrations and incubation time on CCRF-CEM cell viability were investigated to obtain the optimal cellular activity with minimal cell death. Among the tested compounds A, I and 8a, the 8a possessed the most significant anti-proliferative activity and thus was chosen to test the optimal concentrations and treatment time. As shown in Figure 1B, incubation time ranging from 8 to 32 hours did not significantly affect the cytotoxicity of 8a, but its antiproliferative activity increased significantly with increase of the concentrations. At 0.2 µM, 8a was not significantly toxic to CCRF-CEM cells, but led to more than 40% decrease of viable cells when its concentration was increased to 1.0 µM. When 8a was used at 0.5 µM for 24 h, the viability of CCRF-CEM cells was at approximately 80%. Flow cytometry assay confirmed this MTT result that at 0.5 µM for 24 h, 8a led to 16.27% apoptotic cell death (Figure 1C). This treatment condition was used for the following experiments.

### Metabolomic Analysis of Compounds 8a, I and A-treated Cells

**Figure 2:**
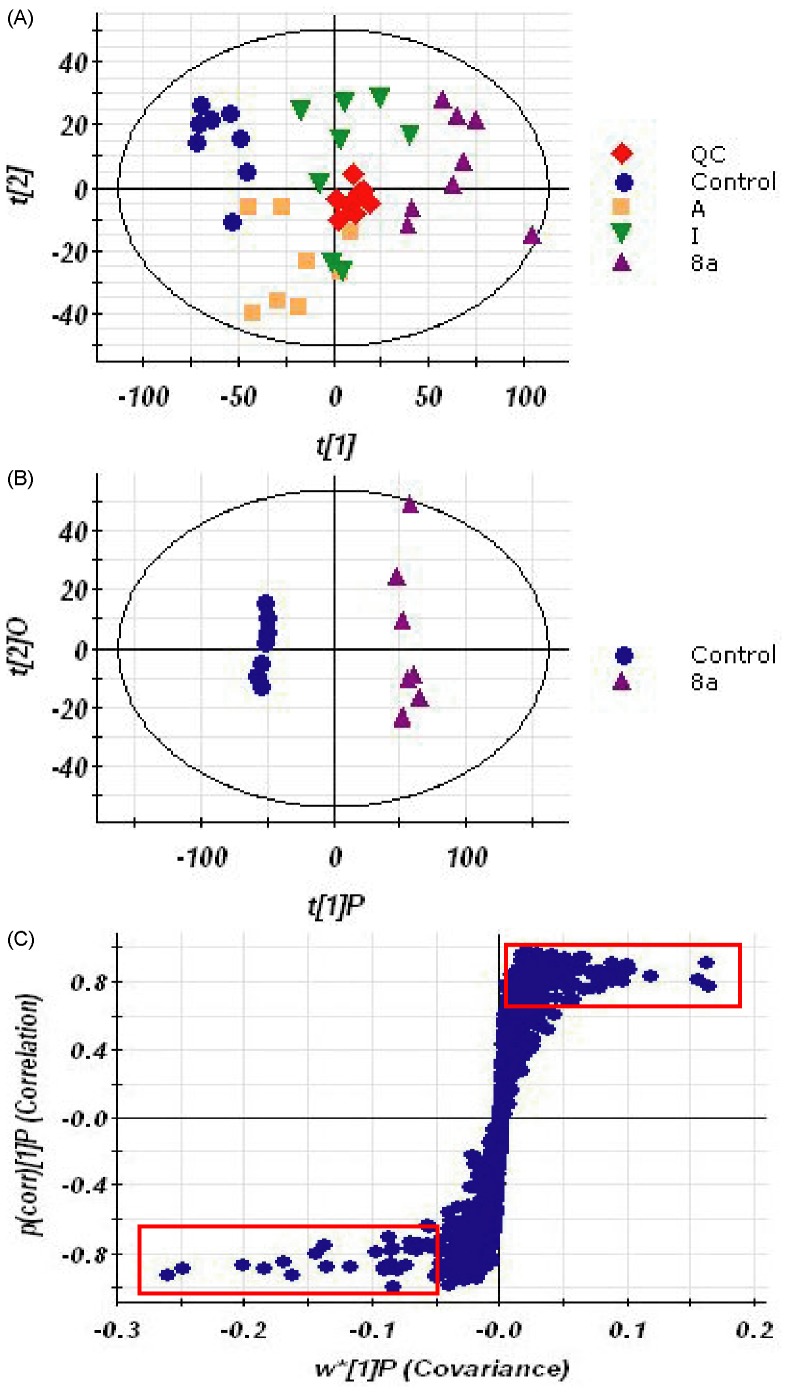
PCA scores plot resulting from the UPLC/MS spectra of CCRF-CEM cells with corresponding OPLS-DA for tested groups. (A) PCA scores plot from control and 8a-treated group. (B) OPLS-DA scores plot from control and 8a-treated group. (C) Potential biomarkers in the S-plot between control and 8a-treated group.

To investigate the metabolic fingerprints in cancer cells, metabolites in CCRF-CEM cells from control and treated groups were profiled by UPLC/Q-TOF MS in both positive and negative modes, though positive ion mode gave more information-rich data than negative. Representative base peak intensity (BPI) chromatograms were shown in Figure S1. Raw data from UPLC/Q-TOF MS were analyzed by the Marker-Lynx software. The acquired QC data for UPLC/Q-TOF MS metabolic profiling were used to investigate the analytical variability in the whole run, which was critical for evaluating the variations in the analytical results and thus the reliability of the metabolite profiling data [37]. As shown in Figure 2A, the QC samples were tightly clustered in PCA scores plot, indicating the non-targeted analysis displayed the column stability in the whole run. Moreover, it was noteworthy that the control and 8a-treated groups were obviously separated along the first principal component, while A and I treated groups were not significantly different from the control group. It is indicated that the cellular metabolic phenotypes were significantly altered under the treatment of 8a. The results were in agreement with aforementioned anti-proliferative activity.

Feature selection was conducted using OPLS-DA for these two groups of cells to depict the metabolic signature. As shown in Figure 2B, the score plots of OPLS-DA between the control and 8a groups were clearly separated. R^2^Y (cum), the fraction of the variation of Y (all the responses) explained by the model after each component, and Q^2^ (cum), the fraction of the variation of Y that can be predicted by the model according to cross-validations, were used to evaluate the OPLS-DA model. The R^2^Y (cum) and Q^2^ (cum) were 0.761 and 0.433, respectively. These results suggest that the model explains 76.1% of the variations of Y, with a predictive ability (Q^2^) of 43.3%. The S-plot is a scatter plot which combines the covariance and correlation loading profiles arising from the predictive components of the OPLS-DA model. We carried out this analysis to visualize variables that have significant contributions to the discrimination of these two groups. The significant variables, with high correlation and covariance values, were located in regions far away from the origin (red boxes in Figure 2C), and were selected as potential biomarkers. An independent t-test indicated that these variables between the control and 8a-treated cells were statistically significant (*p*<0.05).

### Identification of Metabolic Biomarkers

Metabolite identification was conducted with high resolution MS and MS/MS fragments, as well as database analysis. The authentic metabolite standards of interest, as available, were used for confirmation in both *m/z* and RT. Lipids were tentatively identified by high mass accuracy and MS/MS fragment ions without authentic standards comparison [20], [38].

**Figure 3:**
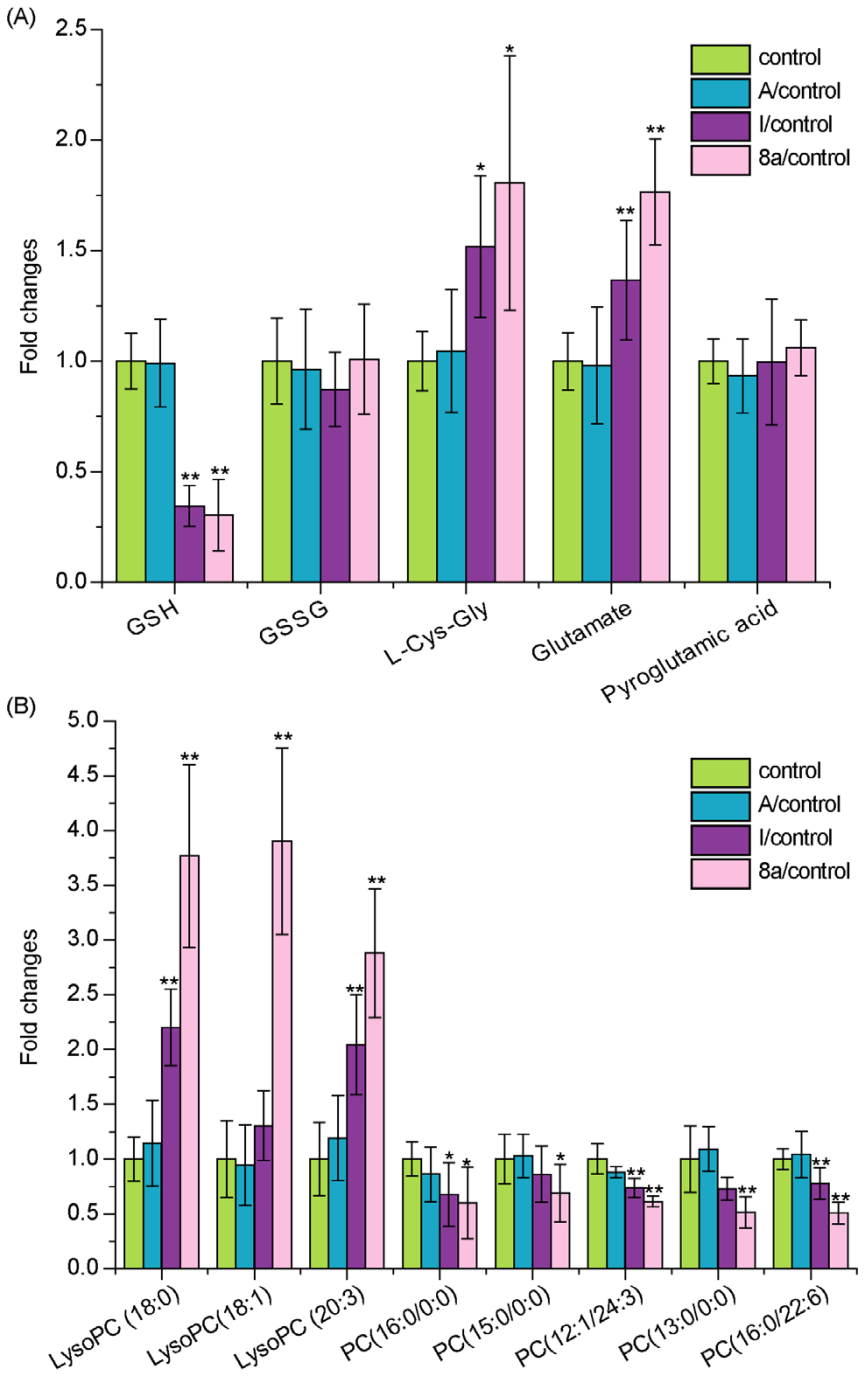
The comparison of biomarker intensity between control and compounds-treated cells. These biomarkers involved in (A) Glutathione metabolism (B) Glycerophospholipid metabolism (n = 8), *p<0.05 and **p<0.01 compared with vehicle control.

On the basis of UPLC/Q-TOF MS metabolomics, identified variables from comparisons (8a, I or A-treated groups vs. control group, respectively) were summarized in Table S2, including RT, the mass obtained in UPLC-MS system, and the mass error when comparing with the database. In addition, the type of the identification (MS/MS fragmentation or confirmation with the analysis of standard), fragment ions of the metabolite, percentage of changes in different comparisons, and statistical significance were also presented in the table. It is shown that the metabolites in 8a-treated group were the most significantly changed. By comparing the 8a-treated group with the control, 23 metabolites were highlighted, which were derived from altered fatty acid, nucleoside, amino acid, glycerophospholipid, and glutathione metabolism. Further studies were focused on 11 metabolites which were involved in glutathione and glycerophospholipid metabolism. As shown in Figure 3, the metabolites in 8a, I and A-treated groups showed the same variation tendency, but extents of the changes in 8a-treated group were significantly larger than the other two groups. Therefore, the analysis of the changed metabolites, as well as the following biological studies, were focused on 8a-treated group.

Metabolites produced in glutathione metabolism include reduced glutathione (GSH), oxidized glutathione (GSSG), L-cysteinyl-glycine (L-Cys-Gly), glutamate, and pyroglutamic acid. GSH, containing an important functional thiol group, is a major endogenous antioxidant and plays a key role in eliminating the free radicals and ROS, as well as reducing oxidized or inactivated protein thiols in order to ensure their biological activities. Therefore, GSH is considered as an important indicator of oxidative stress [39], [40]. As shown in Figure 3A, the reduced GSH was decreased by nearly 70% in 8a-treated cells compared to the vehicle control (8a/control = 0.304±0.161, *p*<0.05), suggesting oxidative stress induced by the acridone derivative 8a. This phenomenon is in accordance with the intoxication condition caused by toxins [41]. Interestingly, the metabolites of GSH, L-Cys-Gly and glutamate, were increased by 80% in the cells treated by 8a (*p*<0.05), suggesting that GSH was also exhausted by increased metabolic degradation. Nevertheless, these results indicate that the novel acridone derivative 8a may induce GSH deficiency and oxidative stress as a primary mechanism for its antitumor activity (see below).

We further investigated glycerophospholipid metabolites with a focus on lysophosphatidylcholines (LPCs) and phosphatidylcholines (PCs) (Figure 3B). Compared to the control, three LPCs, including LysoPC (18∶0), LysoPC (18∶1) and LysoPC (20∶3), were increased by 2.88 times or more in 8a-treated cells. It has been reported that LPCs are produced as oxidation at the sn-2 fatty acids by ROS [42]. These results were consistent with above observations that 8a induced oxidative stress and led to lipid peroxidation. On the contrary, 5 PCs were significantly decreased (*p*<0.05) in 8a-treated cells, including three saturated fatty acid PCs [PC (13∶0/0∶0), PC (15∶0/0∶0) and PC (16∶0/0∶0)] and two unsaturated fatty acid PC [PC (12∶1/24∶3) and PC (16∶0/22∶6)]. These PCs are the main lipid components of biomembranes, and their decrease indicates the lesions of cell membranes induced by 8a [43].

### Metabolites Stability Evaluation

Stability of metabolites is a major concern on the data accuracy of metabolomic studies due to the time-consuming in sample analysis (10–30 min per sample). The stability of metabolites may be influenced by enzymatic action, degradation of macromolecules to release metabolites, and inherent chemical lability. In order to evaluate the metabolites stability, samples were reanalyzed three times with 10 h interval at 4°C to mimic typical autosampler storage. The metabolites peak areas were compared with initial values. As shown in Table S3, the relative standard deviative (RSD) in three independent experiments were less than 7%. These results demonstrated the metabolites tested, including GSH and GSSG, showed excellent stability in the experimental conditions.

### Oxidative Stress and Mitochondrial Lesions Induced by 8a

**Figure 4:**
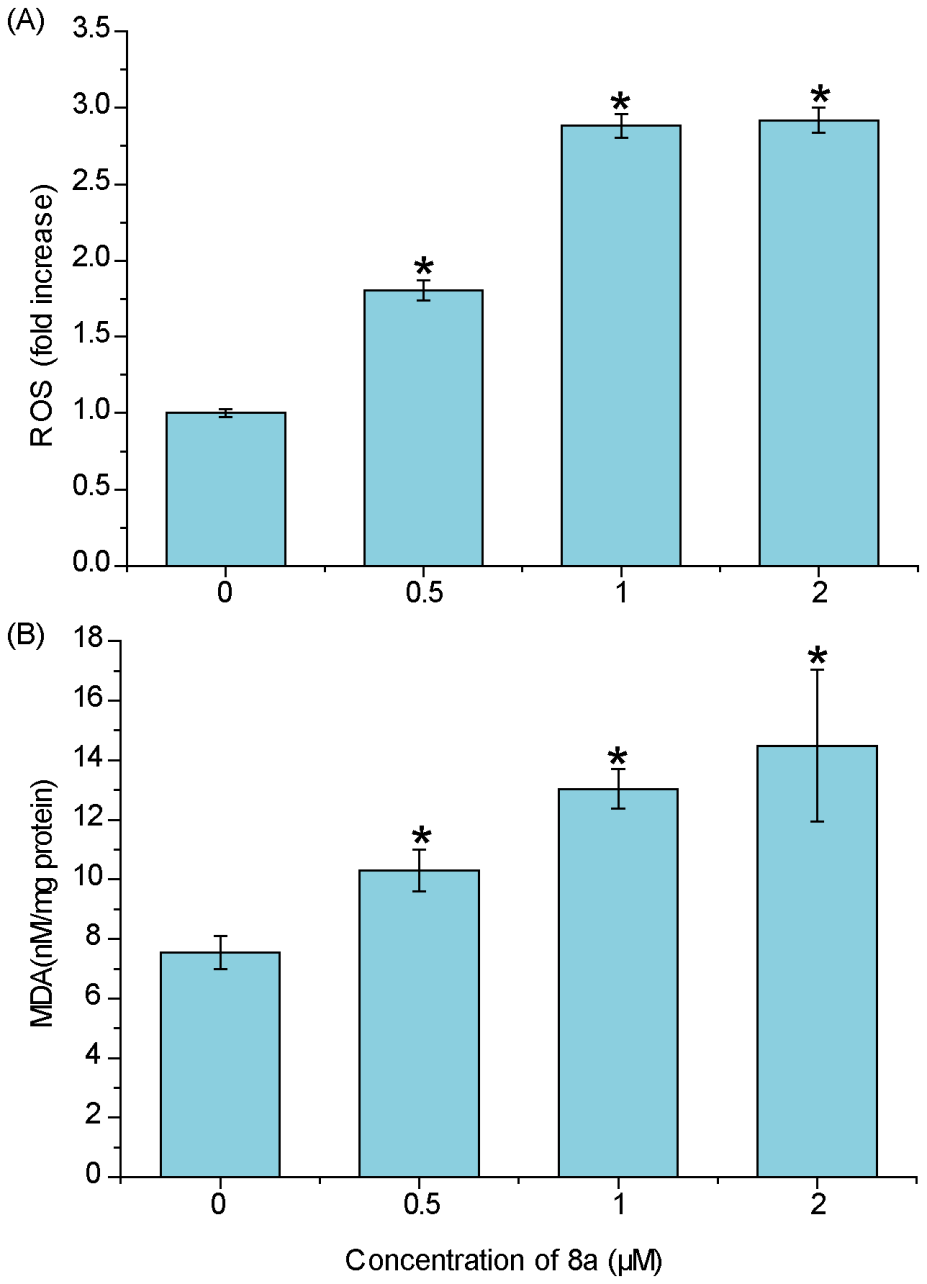
ROS and MDA levels in CCRF-CEM cells. (A) The level of ROS after treatment with compound 8a (0–2 µM) for 24 h. (B) MDA production after 8a (0–2 µM) treatment for 24 h. Data represent the mean±SD, n = 3, *p<0.05 compared with vehicle control.

We further investigated the cellular ROS level and mitochondrial function to prove the oxidative stress and cell death triggered by 8a. The 8a-induced intercellular ROS generation was measured using a permeable fluorescence probe, DCFH-DA, which is oxidized to generate fluorescent DCF in the presence of ROS [44]. DCF fluorescence was detected after cells were exposed to 0.5–2 µM 8a for 24 h. The results showed that the intracellular ROS was significantly increased in a dose-dependent manner (*p*<0.05) (Figure 4A). ROS causes injuries to biomolecules, such as nucleic acids, proteins, structural carbohydrates, and lipids. The peroxidation of lipids by ROS is one of the major injuries to cells. To prove the lipid peroxidation induced by 8a, we tested the cellular MDA levels, a by-product of lipid peroxidation [45]. As shown in Figure 4B, the MDA level was increased by 1.4-fold in the cells treated with 0.5 µM 8a for 24 h, demonstrating a similar tendency to the cellular ROS level. Taking these data together with our aforementioned metabolomic results, it is conclusive that 8a triggers oxidative stress and subsequent lipid peroxidation in the CCRF-CEM cells, leading to the changes of metabolites of GSH and phospholipids.

**Figure 5:**
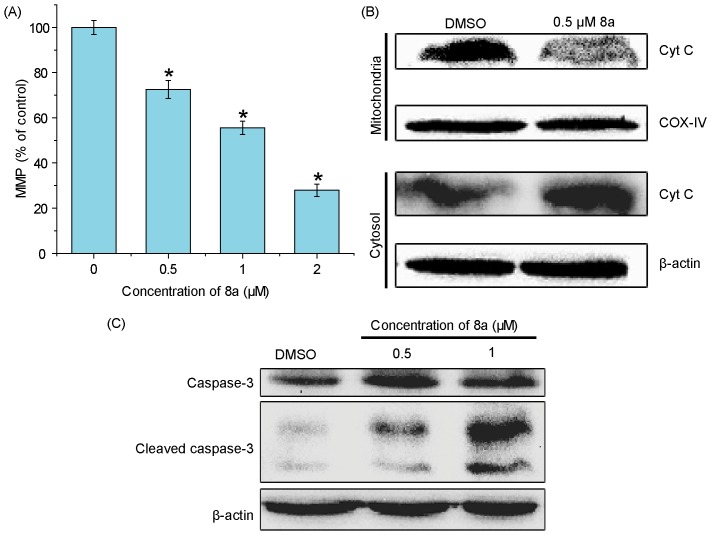
Effects of 8a on the level of MMP and expression of apoptosis-related proteins. (A) MMP affected by 8a in CCRF-CEM cells (n = 3). *p<0.05 compared with vehicle control. (B) Western blot analysis of the effect of 8a on cytochrome C release in CCRF-CEM cells treated with indicated doses of 8a for 24 h. (C) Representative Western blots for the expression of cleaved caspase-3 in CCRF-CEM cells following exposure to different concentrations of 8a for 24 h. β-actin (∼42 kDa) was used as a loading control.

Mitochondria are an important organelle for cell survival and a vulnerable target of ROS. MMP is important to the integrity and function of mitochondria [46], [47]. To understand the cell death mechanism, we further evaluated MMP in 8a-treated cells using a fluorescent dye Rh123, a cell permeable cationic dye which can specifically binds to the active mitochondria based on the highly negative MMP. As shown in Figure 5A, treatment with 0.5–2 µM 8a for 24 h led to drastic decrease of MMP in a dose-dependent manner. When CCRF-CEM cells were exposed to 0.5, 1.0, and 2.0 µM of 8a, the fold changes of MMP to control cells, calculated from the mean MMP of all tested cells, were 0.837±0.039, 0.556±0.029, and 0.279±0.027, respectively.

Intrinsic apoptosis is initiated by a mitochondrial-mediated pathway, including the alteration of MMP, the release of cytochrome C into cytosol, and the activation of caspase-3 [48]. Therefore, we further examined the mitochondrial cytochrome C release. After cells were treated with 8a at 0.5 µM for 24 h, mitochondrial cytochrome C was significantly reduced, accompanied with the increase in cytosol, indicating enhanced cytochrome C release in these cells (Figure 5B). After the reduction of MMP and the release of mitochondrial cytochrome C, a critical step is the formation of apoptosomes, which ultimately cleave procaspase-3 to form active caspase-3. Caspases play critical roles in the execution of apoptosis [49]. Western blot analysis indicated that dramatic caspase-3 was activated in 8a-induced CCRF-CEM cells (Figure 5C). All together, these results suggest a mitochondrial-mediated cell apoptosis pathway is activated by 8a.

### Conclusions

**Figure 6:**
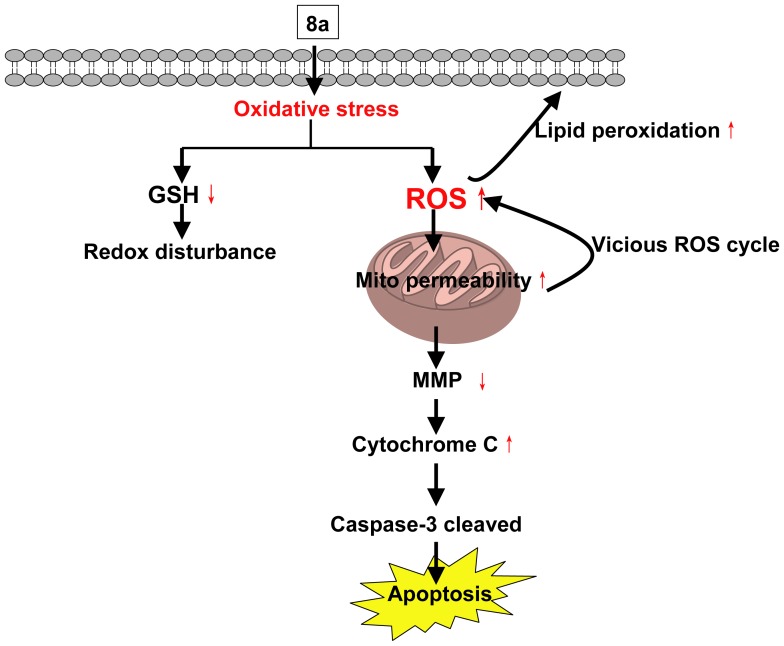
The hypothetic scheme of the action mechanism of 8a. Mito, mitochondrial.

In this study, an UPLC/Q-TOF MS based metabolomic method was established to investigate the metabolite changes of two acridone derivatives (I and 8a) and their parental compound A in CCRF-CEM cells in order to predict hypothetic antitumor mechanism of 8a in CCRF-CEM cells. Results revealed that 8a possessed the strongest anti-proliferative activity and the most significant discrimination in the cell metabolic phenotypes compared with vehicle control group. Comparing 8a with the control group, twenty three distinct metabolites involved in five metabolic pathways were identified. Two metabolic pathways, i.e., glutathione and glycerophospholipid metabolism were investigated in depth to understand the cell death mechanism induced by 8a; 11 metabolites were identified as biomarkers for antitumor activity. Biological studies were further conducted to confirm the underlying cellular events. Combining the metabolomic and biological results, we propose a schematic hypothesis for the anti-proliferative mechanism of 8a (Figure 6). In CCRF-CEM cells, 8a provokes oxidative stress, which in turn leads to lipid peroxidation and mitochondrial lesions and potential changes. Cytochrome C release resulting from mitochondrial potential changes then triggers caspase-3 mediated apoptosis. These findings enhance our understanding of the action mechanisms of 8a anti-proliferation and aid in their incorporation into further improvement.

## Supporting Information

Figure S1 **Metabolomics profile by UPLC/Q-TOF MS. The base peak intensity (BPI) chromatograms obtained from intracellular metabolites of control, A, I and 8a-treated CCRF-CEM cells in (A) ESI+ and (B) ESI− mode.**
(TIF)

Table S1 **Chemical structure and antiproliferative activity against CCRF-CEM cells of compounds A, I and 8a.**
(DOC)

Table S2 **Identification of metabolites connected with glycerophospholipid, glutathione, nucleoside, fatty acid, and amino acids metabolism.** *^a^* Fold change was calculated from the arithmetic mean values of each group. (+): up-regulated. (−): down-regulated compared with controls. *^b^* LysoPC: Lysophosphatidylcholine. *^c^* PC: Lysophosphatidylcholine. *^d^* Metabolites formally identified by standard samples. *^e^* Metabolites putatively annotated.
(DOC)

Table S3 **Variations of 5 metabolites peak area involved in glutathione metabolism.**
(DOC)
