# Supplementary figures and images for: Acridone Derivative 8a Induces Oxidative Stress-Mediated Apoptosis in CCRF-CEM Leukemia Cells: Application of Metabolomics in Mechanistic Studies of Antitumor Agents
Source: PLoS One. 2013 May 7;8(5):e63572. doi: 10.1371/journal.pone.0063572 (PMC3646819; doi:10.1371/journal.pone.0063572)

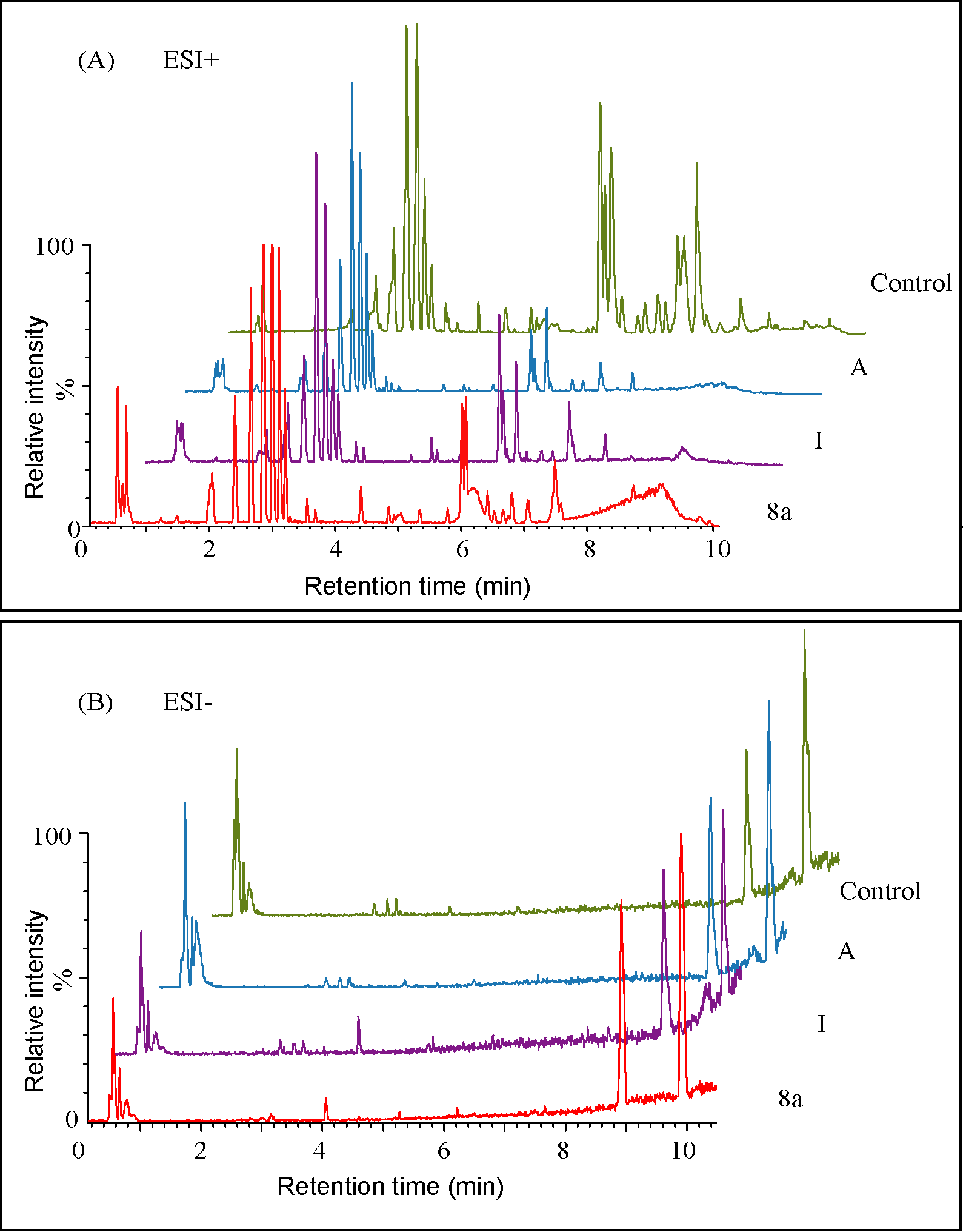

Supplement: Figure S1 — Metabolomics profile by UPLC/Q-TOF MS. The base peak intensity (BPI) chromatograms obtained from intracellular metabolites of control, A, I and 8a-treated CCRF-CEM cells in (A) ESI+ and (B) ESI− mode. (TIF) [file pone.0063572.s001.tif]
