# Supplementary material for: Acridone Derivative 8a Induces Oxidative Stress-Mediated Apoptosis in CCRF-CEM Leukemia Cells: Application of Metabolomics in Mechanistic Studies of Antitumor Agents
Source: PLoS One. 2013 May 7;8(5):e63572. doi: 10.1371/journal.pone.0063572 (PMC3646819; doi:10.1371/journal.pone.0063572)
Supplement: Table S1 — Chemical structure and antiproliferative activity against CCRF-CEM cells of compounds A, I and 8a. (DOC) [file pone.0063572.s002.doc]

**Table S1.** Chemical structure and antiproliferative activity against CCRF-CEM cells of compounds A, I and 8a.

| Abbreviation | Chemical name | Chemical structure | IC_50_*^a^* (μM) |
| --- | --- | --- | --- |
| A | 9(10H)-acridone | 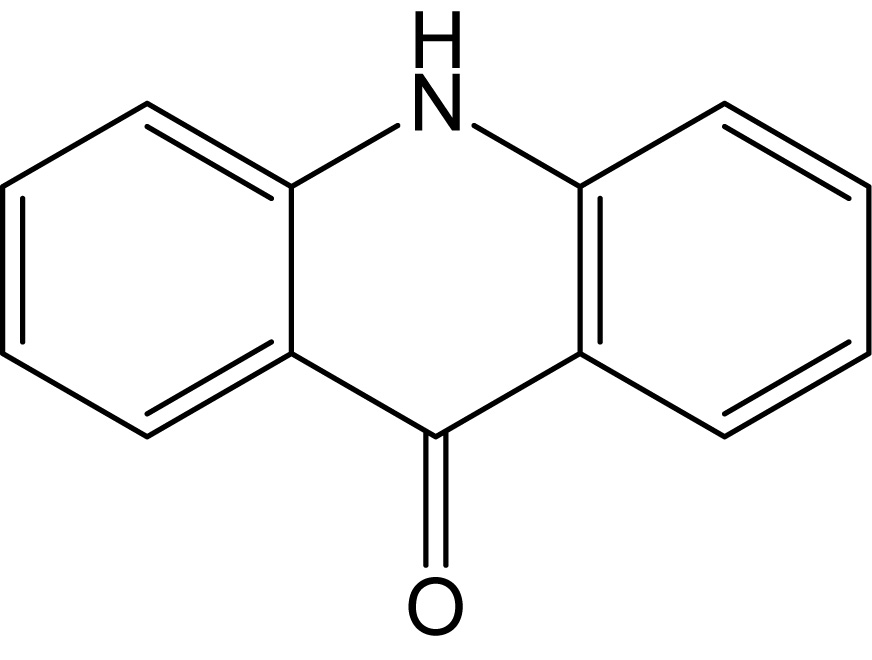 | >100 |
| I | 10-(3,5-dimethoxy)benzyl-9(10H)-acridone | 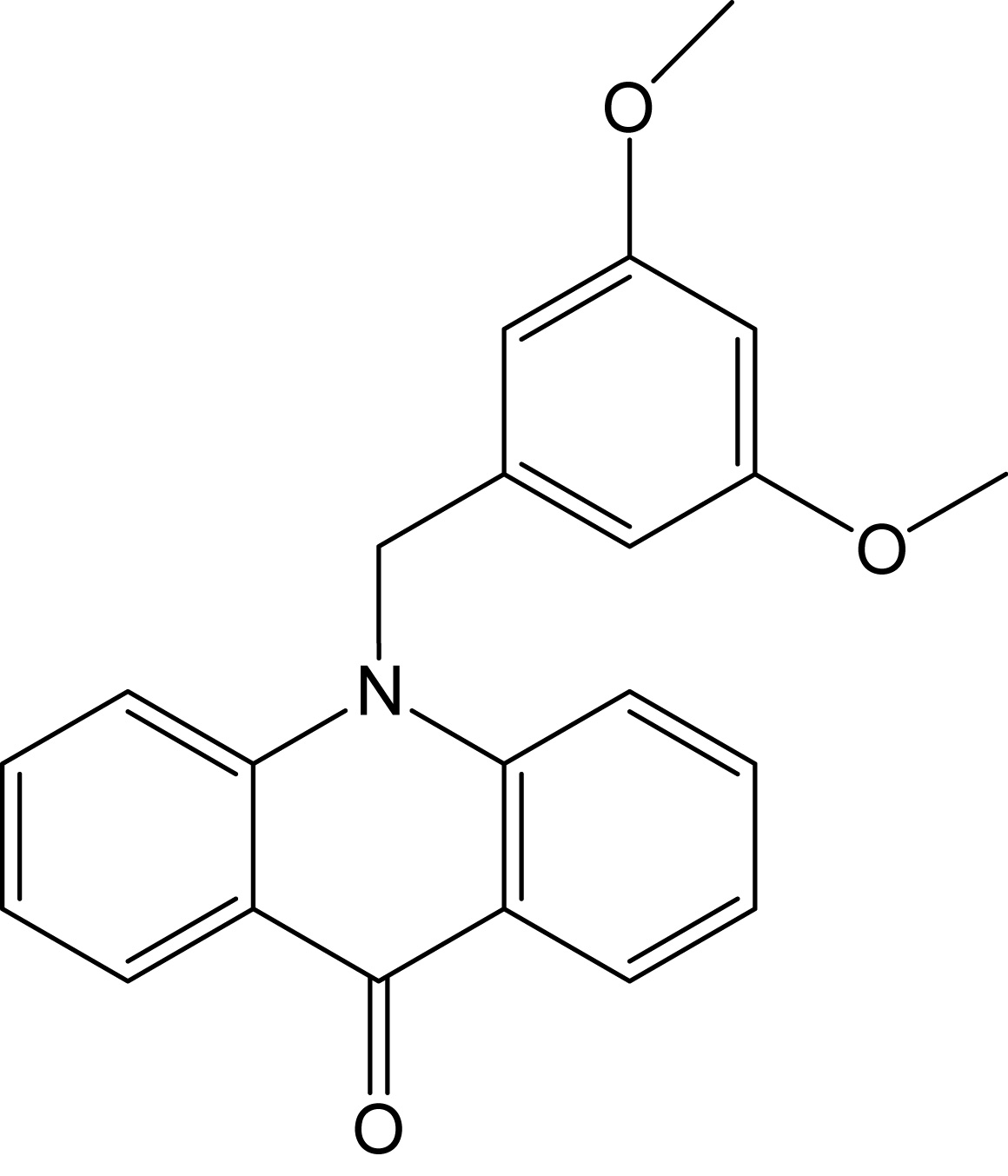 | 0.7 |
| 8a | 2-aminoacetamido-10-(3, 5-dimethoxy)-benzyl-9(10H)-acridone hydrochloride | 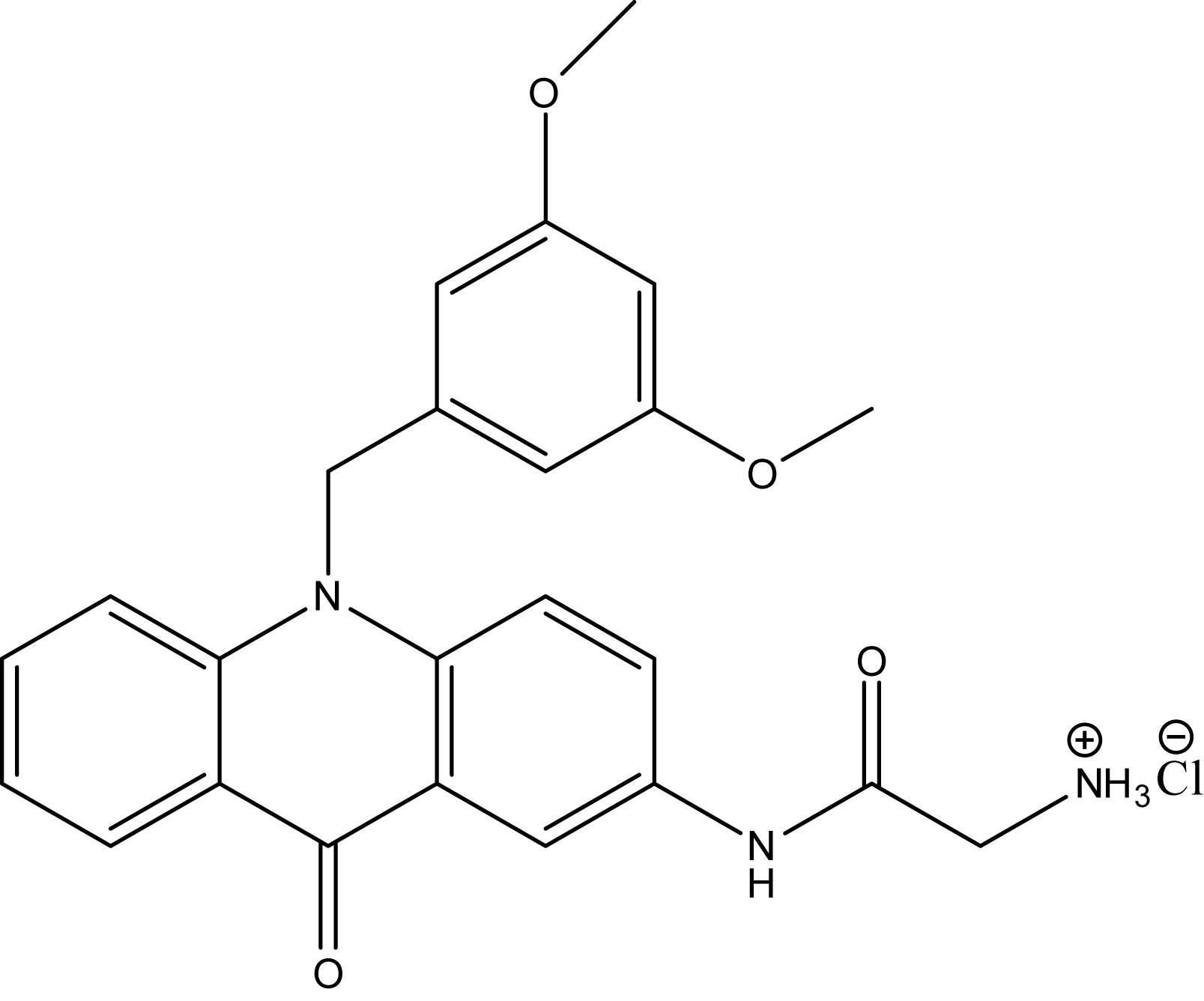 | 0.39 |

*^a^* IC50 values were determined from MTT proliferation assays after incubation with test compound for 48 h.
