# Supplementary material for: Acridone Derivative 8a Induces Oxidative Stress-Mediated Apoptosis in CCRF-CEM Leukemia Cells: Application of Metabolomics in Mechanistic Studies of Antitumor Agents
Source: PLoS One. 2013 May 7;8(5):e63572. doi: 10.1371/journal.pone.0063572 (PMC3646819; doi:10.1371/journal.pone.0063572)
Supplement: Table S2 — Identification of metabolites connected with glycerophospholipid, glutathione, nucleoside, fatty acid, and amino acids metabolism. a Fold change was calculated from the arithmetic mean values of each group. (+): up-regulated. (−): down-regulated compared with controls. b LysoPC: Lysophosphatidylcholine. c PC: Lysophosphatidylcholine. d Metabolites formally identified by standard samples. e Metabolites putatively annotated. (DOC) [file pone.0063572.s003.doc]

**Table S2.** Identification of metabolites connected with glycerophospholipid, glutathione, nucleoside, fatty acid, and amino acids metabolism. *^a^* Fold change was calculated from the arithmetic mean values of each group. (+): up-regulated. (-): down-regulated compared with controls. *^b^* LysoPC: Lysophosphatidylcholine. *^c^* PC: Lysophosphatidylcholine. *^d^* Metabolites formally identified by standard samples. *^e^* Metabolites putatively annotated.

| Compound | RT (min) | Measured mass (Da) | Mass error (ppm) | Fragment ions | Change(%)*^a^* *p-*value | | |
| --- | --- | --- | --- | --- | --- | --- | --- |
|  |  |  |  |  | A | I | 8a |
| Choline*^d^* | 0.53 | 104.1049 | -16.3 | 58.1,60.1,56.1 | 49 (3.04E-07) | 79 (1.18E-05) | 91 (2.27E-06) |
| Acetylcholine*^d^* | 0.58 | 146.1162 | -10.2 | 64.1,87.0, | -1 (0.94) | -53 (0.01) | -54 (3.41E-03) |
| Phosphocholine*^d^* | 0.56 | 184.0733 | 1.6 | 86.1,99.0,125.0,166.1 | -14 (0.18) | -18 (0.08) | -35 (2.67E-03) |
| LysoPC*^b^*(20:3)*^e^* | 7.71 | 546.3678 | -21.7 | 104.1, 184.1, 528.4 | 19 (0.31) | 105 (1.28E-04) | 188 (1.67E-06) |
| LysoPC(18:1)*^e^* | 7.03 | 522.3512 | -8.0 | 104.1, 184.1, 504.3 | -19 (0.58) | 30 (0.55) | 290 (2.58E-04) |
| LysoPC (18:0)*^e^* | 7.71 | 524.3762 | 9.3 | 104.1, 184.1,506.4 | 14 (0.37) | 120 (7.63E-07) | 277 (1.91E-05) |
| PC*^c^*(16:0/0:0)*^e^* | 6.85 | 496.3397 | 0.2 | 104.1, 184.1,478.3 | -14 (0.23) | -32 (0.02) | -40 (0.01) |
| PC(15:0/0:0)*^e^* | 7.09 | 482.3641 | 7.3 | 86.2,104.1,184.1,258.3,464.3 | 3 (0.78) | -14 (0.28) | -31 (0.03) |
| PC(13:0/0:0)*^e^* | 7.18 | 454.2931 | -0.4 | 104.1, 184.11,436.3 | 9 (0.49) | -27 (0.05) | -49 (7.21E-03) |
| PC(12:1/24:3)*^e^* | 9.30 | 782.5790 | 11.5 | 104.1, 184.1,438.2 | -12 (0.06) | -26 (5.71E-04) | -39 (4.23E-06) |
| PC(16:0/22:6)*^e^* | 9.33 | 806.5691 | -0.4 | 104.1, 184.1,496.3 | 4 (0.63) | -22 (3.71E-03) | -49 (4.57E-04) |
| Glutathione*^d^* | 0.62 | 308.0893 | -13.3 | 84.0,116.0,162.0,179.0,233.0 | -1 (0.95) | -66 (5.61E-04) | -70 (8.65E-05) |
| Glutathione, oxidized*^d^* | 0.61 | 613.1574 | -4.7 | 231.0,355.1,484.1 | -24 (0.06) | -13 (0.18) | -10 (0.37) |
| Pyroglutamic acid*^d^* | 0.56 | 130.0496 | -0.7 | 57.2,64.0,84.0,101.1 | -7 (0.35) | 0 (0.96) | 6 (0.33) |
| L-Cys-Gly*^d^* | 0.61 | 179.0505 | 16.7 | 76.0,144.2,163.0 | 35 (0.25) | 52 (0.05) | 81 (0.03) |
| Glutamate*^d^* | 0.56 | 148.0602 | 0.6 | 84.1，85.0,130.1 | -2 (0.86) | 37 (3.87E-03) | 77 (1.49E-06) |
| Hypoxanthine*^d^* | 0.61 | 137.0454 | -0.7 | 119.0,110.1,94.1 | 723 (4.55E-03) | 1050 (1.55E-03) | 1695 (4.11E-05) |
| Uridine*^d^* | 0.56 | 243.0632 | 10.2 | 82.0, 110.0, 153.0, 200.0 | 36 (0.23) | 203 (3.18E-04) | 67 (0.01) |
| Inosine*^d^* | 0.56 | 267.0718 | -0.4 | 135.0，149.3,267.1 | 85 (3.90E-03) | 398 (6.84E-05) | 631 (2.59E-03) |
| Palmitoyl-L-carnitine*^d^* | 7.14 | 400.3443 | 7.5 | 85.0,341.3,239.3 | 2 (0.90) | -34 (0.10) | -36 (0.01) |
| Palmitic acid*^d^* | 8.85 | 255.2316 | -3.9 | standard | 46 (0.02) | 64 (0.01) | 68 (0.01) |
| L-Phenylalanine*^d^* | 0.56 | 164.0715 | 2.4 | 72.0, 103.1, 147.1 | -18 (0.17) | 2 (0.91) | -41 (0.02) |
| Pantothenic acid*^d^* | 0.57 | 218.0997 | -9.2 | 88.0，146.0 | -68 (3.78E-05) | -44 (2.93E-03) | -72 (6.81E-06) |
