# Supplementary material for: Acridone Derivative 8a Induces Oxidative Stress-Mediated Apoptosis in CCRF-CEM Leukemia Cells: Application of Metabolomics in Mechanistic Studies of Antitumor Agents
Source: PLoS One. 2013 May 7;8(5):e63572. doi: 10.1371/journal.pone.0063572 (PMC3646819; doi:10.1371/journal.pone.0063572)
Supplement: Table S3 — Variations of 5 metabolites peak area involved in glutathione metabolism. (DOC) [file pone.0063572.s004.doc]

**Table S3.** Variations of 5 metabolites peak area involved in glutathione metabolism.

| metabolite | Glutathione | Glutathione, oxidized | Pyroglutamic acid | L-Cys-Gly | Glutamate |
| --- | --- | --- | --- | --- | --- |
| Peak area | 1038.6 | 1028.5 | 396.8 | 274.9 | 436.5 |
| RSD (%) | 5.596 | 5.099 | 4.897 | 6.461 | 5.054 |
